# Tumour neoantigen mimicry by microbial species in cancer immunotherapy

**DOI:** 10.1038/s41416-021-01365-2

**Published:** 2021-04-06

**Authors:** Maximilian Boesch, Florent Baty, Sacha I. Rothschild, Michael Tamm, Markus Joerger, Martin Früh, Martin H. Brutsche

**Affiliations:** 1 Lung Center, Cantonal Hospital St. Gallen, St. Gallen, Switzerland; 2 Department of Medical Oncology and Comprehensive Cancer Center, University Hospital of Basel, Basel, Switzerland; 3 Department of Pulmonology, University Hospital of Basel, Basel, Switzerland; 4 Department of Medical Oncology and Hematology, Cantonal Hospital St. Gallen, St. Gallen, Switzerland; 5 Department of Medical Oncology, University Hospital Bern, Bern, Switzerland

**Keywords:** Tumour immunology, Tumour immunology

## Abstract

Tumour neoantigens arising from cancer-specific mutations generate a molecular fingerprint that has a definite specificity for cancer. Although this fingerprint perfectly discriminates cancer from healthy somatic and germline cells, and is therefore therapeutically exploitable using immune checkpoint blockade, gut and extra-gut microbial species can independently produce epitopes that resemble tumour neoantigens as part of their natural gene expression programmes. Such tumour molecular mimicry is likely not only to influence the quality and strength of the body’s anti-cancer immune response, but could also explain why certain patients show favourable long-term responses to immune checkpoint blockade while others do not benefit at all from this treatment. This article outlines the requirement for tumour neoantigens in successful cancer immunotherapy and draws attention to the emerging role of microbiome-mediated tumour neoantigen mimicry in determining checkpoint immunotherapy outcome, with far-reaching implications for the future of cancer immunotherapy.

## Background

One of the hallmarks of cancer is the overly high proliferative capacity of tumour cells, which can be therapeutically exploited using classical cytotoxic chemotherapy. The merit of anti-proliferative drugs in the debulking of large tumours is beyond dispute, but these agents are molecularly unselective for cancer, and invariably also damage healthy tissue such as bone marrow and the colonic mucosa.^1^ Targeted drugs have successfully tackled this issue and facilitated cancer-predominant therapy with a superior safety profile (e.g. trastuzumab for HER2^+^ breast cancer,^2^ crizotinib for EML4-ALK^+^ lung cancer,^3^ imatinib for BCR-ABL^+^ leukaemia^4^). However, most targeted drugs are not exclusively specific for cancer, because they usually inhibit wild-type versions of proteins, which can often be expressed in a wide range of tissues (e.g. HER2 is also expressed in cardiomyocytes,^5^ and crizotinib^6^ and imatinib^7^ inhibit wild-type ALK and ABL tyrosine kinases, respectively, as well as the underlying cancer-specific gene fusions), and some small-molecule inhibitors, particularly first-generation agents, are multi-specific, thus showing additional on-target activity (e.g. crizotinib also inhibits MET and ROS1,^8,9^ and imatinib also inhibits c-kit and platelet-derived growth factor receptor^10^). Chimeric antigen receptor T cells also lack molecular specificity for cancer as their engineered affinities generally recognise wild-type antigens expressed on non-transformed cells as well as transformed cells, potentially leading to on-target, off-tumour toxicity (e.g. CD19 is expressed on both the malignant and healthy B-cell lineage).^11,12^

Despite the growing appreciation of epigenetic regulation^13^ and well-documented evidence for infectious contributions to tumour development (e.g. human papilloma virus^14^ and *Helicobacter pylori*^15^), the dogma that ‘cancer is a genetic disease’ still holds true.^16^ The stepwise accumulation of mutations not only facilitates and exacerbates malignancy, but also generates a molecular fingerprint—comprised of tumour neoantigens—with definite specificity for cancer. While some tumour neoantigens are recurrent among cancer entities (e.g. KRAS position 12 mutations), the overall molecular fingerprint of a cancer is unique in every patient irrespective of tumour type. This collective of cancer-specific molecular aberrations, commonly termed the ‘tumour neoantigenome’, is an ideal target for cell-mediated immunity and cancer immunotherapy, with arguably the highest possible specificity for cancer considering the natural selectivity of cytotoxic, adaptive immune cells.^17–19^ Exogenously derived antigens can also be regarded as ‘tumour neoantigens’^20^ but, compared with endogenous neoantigens, the presence of viral or bacterial antigens is limited to relatively few cancer entities (e.g. cervical cancer, gastric cancer, hepatocellular carcinoma, head and neck squamous cell carcinoma (HNSCC)).^21^ In addition, infection-related cancers still carry endogenous neoantigens,^22–24^ and some of these malignancies can be prevented with vaccines.^25^ This article will therefore prioritise endogenous tumour neoantigens arisen from mutations in host cells.

Although the presence of tumour neoantigens can discriminate cancer cells from ‘normal’ cells, epitopes produced by gut and extra-gut microbial species as part of their natural gene expression programmes can resemble tumour neoantigens—a phenomenon known as molecular mimicry. Molecular mimicry of tumour neoantigens by microbial species is likely to influence the host anti-cancer immune response through neoantigen-reactive T cells, and could explain why certain patients show favourable long-term responses to immune checkpoint blockade while others do not benefit at all from this treatment—tumour neoantigens are a mechanistic requirement for successful cancer immunotherapy.

In this article, we will introduce the concept of microbial tumour neoantigen mimicry emanating from the huge overall amount and diversity of microbial genetic sequences in the human body, and elaborate on the potential significance of such mimicry for checkpoint immunotherapy responsiveness and outcome.

## Tumour neoantigens

Tumour neoantigens mostly constitute de novo mutations that arise in transformed cells when tumour DNA is altered through non-synonymous point mutations, gene translocations, or insertions and deletions (indels)^20^ and are excluded from central tolerance^20^—that is, they are not included in the mechanism used by the host to avoid attack by the immune system. Tumour neoantigens can also, in some cases, arise from the cancer-cell-specific usage of the epigenetic machinery, which can lead to the re-expression of silenced genes, the occurrence of unique splice or structural variants, and/or read-through transcription generating chimeric or intergenic products.^20,26,27^

## Immunological visibility and immune evasion

Generally, immunological visibility of tumour neoantigens requires protein synthesis and a certain stability of the neoantigen product. Following the release of cytoplasmic content during cell death, for example, tumour neoantigens expressed as proteins will be taken up by tissue-patrolling antigen-presenting cells (APCs), which will then migrate to the tumour-draining lymph node and present the molecular tumour fingerprint to CD8^+^ naive T cells (cross-priming).^28–30^ In turn, interactions between T cells with a cognate T cell receptor and the neoantigen-positive APCs induce clonal expansion and effector/memory differentiation;^31^ cytotoxic tumour killing by neoantigen-specific T cells results in cancer cell lysis, which might generate an ‘in situ vaccination effect’ such that beneficial epitope spreading might occur.^32^ In an ideal scenario, sustained anti-cancer immunity will ensue protection from cancer re-growth.

However, clinical evidence indicates that such protective anti-cancer immunity is rare among cancer patients, owing to immune evasion caused by various cancer-cell-intrinsic^33–35^ and microenvironmental mechanisms.^1,36^ In terms of cancer-cell-intrinsic mechanisms, downregulation of the major histocompatibility complex class I (MHC I) presentation pathway, which is essential for immune recognition and CD8^+^-cytotoxic-cell-mediated killing, is often observed in tumour cells, as is the increased expression of immune checkpoint molecules, which function as a ‘brake’ on the immune system, controlling the duration and extent of immune responses, maintaining self-tolerance and preventing autoimmunity.

## Immune checkpoint inhibitors

Immune checkpoint inhibitors (ICIs) that target checkpoint components, such as programmed death receptor (PD-1) and its ligand programmed cell death ligand 1 (PD-L1), or cytotoxic T-lymphocyte-associated protein 4 (CTLA-4), or other inhibitory molecules can partly release this immune system brake and have shown impressive long-term outcomes in many cancer patients.^37–40^ Mechanistically, ICIs neutralise negative feedback from immunological checkpoints or increase immune-stimulatory signalling, thus reinvigorating the endogenous tumour-specific T cell response. For CTLA-4-targeted immunotherapy, it has been shown that the clinical mode of action is likely to involve therapy-enhanced T cell priming, which broadens the tumour-specific CD8^+^ T cell response.^41^ Data from anti-CTLA-4-treated melanoma patients demonstrated a significant increase in the number of newly detected tumour-specific CD8^+^ T cell responses after treatment, whereas pre-existing virus- and tumour-specific T cell responses remained unchanged.^41^ Similar findings were reported for ICIs that target PD-1, thus altogether suggesting that pre-existing tumour-reactive T cells have a limited reinvigoration potential, which is outcompeted by de novo arising T cell specificities (clonal replacement).^42^ The poor reinvigoration capacity of pre-existing tumour-specific T cells might indicate a partially exhausted cell state maintained independently from checkpoint signalling, which potentially limits the use of ICIs in certain patients.

The clinical significance of ICIs is tremendous, as these agents have shown unprecedented responses even in advanced-stage tumours,^43^ and have established themselves as frontline therapeutics in a broad variety of solid tumour types.^44,45^ However, the speed of further development of ICI-based immunotherapy has started to decelerate, and significant challenges lie ahead, including the need for adapted clinical trial design and customisation of clinical safety and efficacy endpoints to more specifically account for the characteristic features of ICIs compared with classical anti-cancer drugs.^46^

## The relevance of tumour neoantigens in medical oncology

The relevance of tumour neoantigens in medical oncology is highlighted by the positive association between tumour mutational burden (TMB) and the response to ICI treatment.^47–53^ Accordingly, tumour entities with a typically high TMB (e.g. lung cancer, skin cancer, microsatellite-instable cancers) respond significantly better to ICI-based immunotherapy than do cancers with a comparatively low TMB (e.g. breast cancer, prostate cancer).^54^ Moreover, immunological treatments that target the tumour neoantigenome through reinvigoration (in the case of ICIs) or induction (for vaccines) of antigen-specific CD8^+^ T cells do not rely solely on the ‘driver’ nature of mutations, as passenger mutations can be equally immunogenic and thereby also serve as targets for immune destruction;^19,55,56^ by contrast, targeted treatment options require that the respective target molecule is causal for tumour growth and/or progression (‘oncogene addiction’).^57,58^

Thus, tumour neoantigens arising from cancer-specific mutations are attractive targets for cancer immunotherapy, which has shown proven clinical relevance and important conceptual advantages over other types of cancer treatment that do not exploit the natural adaptability and versatility of the immune system.

## Checkpoint blockade responsiveness

Although ICIs have revolutionised the treatment of various solid tumour types and enabled unprecedented long-term remissions, a significant number of patients do not benefit from these therapies, owing to primary, adaptive, or acquired resistance mechanisms^59,60^ shown by tumours. Primary resistance mechanisms include the absence of a relevant number of immunogenic tumour antigens, defects in the antigen processing and presenting machinery, and insufficient T cell infiltration.^59^ Adaptive resistance to cancer immunotherapy refers to mechanisms by which cancer cells adapt to attacking immune cells, for instance through downregulation of MHC class I or upregulation of PD-L1.^59^ Acquired resistance refers to a clinical scenario where a patient initially responds to immunotherapy but later on progresses or relapses; oftentimes, this will involve Darwinian selection of resistant clones present already before treatment start.^59^

Only a small proportion of patients with non-small cell lung cancer (NSCLC), for example, respond favourably to ICI treatment.^61–63^ The time lost in treating poorly responding or refractory patients, along with the potential toxicity^64,65^ and high associated costs of ICI treatment,^66,67^ demands that better tools and predictive markers are established to discriminate patients who are likely to respond to treatment from those who are not likely to respond. More importantly, identifying the underlying mechanisms of non-responsiveness will be instrumental for the development of therapies that work in combination with ICIs to resensitise tumours to checkpoint inhibition.

## Current markers for predicting responsiveness

Aside from TMB as a surrogate indicator for tumour neoantigens, the extent of tumour T cell infiltration at baseline and the expression of the respective ICI target (e.g. PD-1/PD-L1) at the protein level represent the most established markers for predicting the responsiveness to checkpoint blockade.^50,68,69^ However, none of these markers is perfect and they are currently unable to predict treatment response with clinically sufficient precision.^70,71^ As an example, the predictive power of baseline tumour T cell infiltration might be reduced or lost if these T cells are exhausted or otherwise non-functional. Similarly, a high TMB indicating increased statistical odds for the presence of tumour neoantigens does not predict whether these cancer-specific antigens are immunogenic to an extent that is therapeutically relevant. Finally, the proven presence of the ICI target protein only indicates that the substrate is present to inhibit, but not whether, or to what extent, this substrate is causal for tumour growth. Therefore, novel and more reliable predictive markers are desirable to refine patient stratification and further optimise ICI treatment.

## The significance of the gut microbiome in the responsiveness to ICI

Within the past decade, evidence has suggested an important role for the gut microbiome in determining the responsiveness to checkpoint immunotherapy. Two articles published in 2015 demonstrated that commensal *Bifidobacterium*^72^ and distinct *Bacteroides* species^73^ can promote anti-cancer immunity during ICI treatment. Accordingly, co-treatment with antibiotics reduces the clinical activity of checkpoint immunotherapy,^74–78^ whereas faecal microbiome transplantation (FMT) from ICI-responding patients to germ-free or antibiotic-treated mice partly rescues the anti-cancer effects of ICI treatment, dependent on the presence of *A. muciniphila* Gram-negative bacteria.^79^ Although these data can offer possibilities for straightforward therapeutic development (e.g. oral supplementation with relevant bacterial strains, FMT from responding patients), the mechanisms responsible for these observations remain elusive and a deeper understanding of the underlying biology is desirable.^80^

## Immunological mimicry of tumour neoantigens by microbial peptide products in ICI responsiveness

**Fig. 1:**
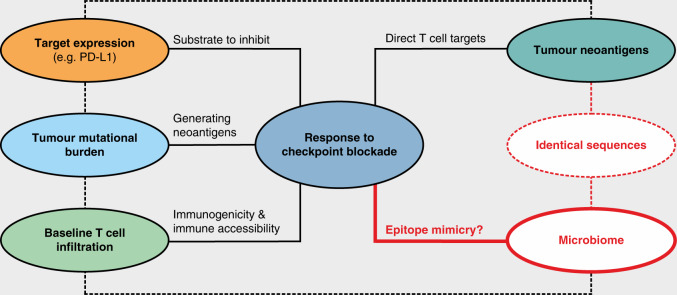
Understanding checkpoint immunotherapy responsiveness. Depicted are important clinical parameters known to influence the response to checkpoint-based immunotherapy. The expression of the targets of immune checkpoint inhibitors (ICIs), the level of tumour mutational/neoantigen burden and the extent of T cell infiltration at baseline are established clinical predictors of ICI treatment response; the molecular contribution of the (gut) microbiome, however, is less well-studied. Epitope mimicry of tumour neoantigens is likely to explain the functional importance of microbial species in checkpoint immunotherapy responsiveness and arguably deserves prioritisation for scientific elaboration efforts. Note the interdependence of the various checkpoint-predictive markers and parameters, indicated with dashed lines. In brief, cancer cell-specific mutations are a requirement for tumour neoantigens, the number of which is thought to correlate with the overall tumour mutational burden. Both tumour neoantigens and tumour mutational burden influence the infiltration of tumours by T cells and are further associated with checkpoint molecule expression. The host microbiome also modulates the response to checkpoint blockade and is connected with tumour neoantigens through molecular mimicry. PD-L1, programmed cell death ligand 1.

A likely explanation for the functional significance of the gut microbiome in checkpoint immunotherapy responsiveness is immunological mimicry of tumour neoantigens by microbial peptide products^81–85^ (Fig. 1). Support for this hypothesis comes from the observation that the efficacy of anti-CTLA-4 treatment depends on antigen-specific immune reactivity against certain bacterial strains, including *B. fragilis*. Accordingly, if *B. fragilis* is experimentally lacking (in germ-free or antibiotic-treated mice), anti-CTLA-4 treatment efficacy can be restored by oral gavage of *B. fragilis*, immunisation with *B. fragilis*-derived products or adoptive transfer of T cells specific for *B. fragilis*.^73^ It is worth mentioning in this context that a 2020 publication on cancer immunotherapy targeting CD47 (another checkpoint molecule) found that the accumulation of gastrointestinal bacteria in subcutaneously grown tumours was critical for the treatment response.^86^ In addition, another study showed that intratumoural accumulation of bacteria is common among cancer entities, with bacterial compositions being mostly biased towards intracellular bacteria that are capable of triggering CD8^+^ T cell responses.^87^ These results highlight the far-reaching effects of gastrointestinal microbes across organ systems/compartments and clearly emphasise the significance of the local tumour microbiome for responsiveness to cancer immunotherapy. Aside from the well-known gastrointestinal microbiome, a rich flora of microbial communities can also be found in the respiratory tract^88,89^ as well as on other various mucosal surfaces and non-mucosal surfaces of the human body.^90–92^ Thus, certain carcinomas (e.g. NSCLC, HNSCC, cervical cancer) are likely to be influenced by a distinct tumour-associated habitat that might also shape the response to ICI therapy.

Convincing evidence therefore suggests that the outcome of ICI-based cancer immunotherapy is influenced, at least partially, by the composition and functional quality of the (distant) gut- and the (local) tumour microbiome.

## Tumour neoantigens and tumour neoantigen mimicry

Cancer immune surveillance is, to a large extent, carried out by CD8^+^ T cells. Molecularly, these cells recognise tumour neoantigens typically as 8–11-mer peptides^93,94^ bound to MHC class I on the tumour cell surface. Random 11-mer permutation of the 20 proteinogenic amino acids in humans (not counting atypical amino acids such as selenocysteine) can generate more than 10^14^ theoretical combinations, although the number of possible tumour neoantigens is correspondingly smaller considering that many of these sequence combinations will already be part of the host genome.

### How does microbial mimicry to tumour antigens arise?

Many microbes that colonise the human body can synthesise all 20 proteinogenic amino acids of humans,^95,96^ and viruses can harness the host cell translational machinery to exclusively use the endogenous amino acid repertoire. It is believed that the human gastrointestinal tract harbours approximately 10^14^ microbes, which, together, equate to roughly 1000 times the number of cells and 10000 times the DNA content of the human body.^97^ Exact mathematical modelling is beyond the scope of this article, but it is with definite statistical likelihood that numerous individual tumour neoantigens are immunologically mimicked by peptide products of gut and extra-gut microbial species: identical peptide mimics comprise the same amino acid sequence as the corresponding tumour neoantigens and result in identical T cell epitopes, whereas ‘notably similar’ peptide mimics contain the same epitope core but differ in their flanking region or length, thus altering antigen presentation efficiency and/or generating T cell epitopes with nuance-like specificity alterations.^98,99^ Importantly, the actual likelihood of mimicry might be smaller than theoretically possible considering that microbial genomes encode functional proteins whose sequences might not be randomly structured which might lower the number of actually possible sequence permutations. In addition, not every neoantigen-mimicking microbial peptide will reach a concentration that is high enough for immunological recognition and modulation of the anti-cancer immune response.

### The role(s) of tumour neoantigen microbial mimicry

The abundance of class-switched IgA antibodies with high affinities for microbial species in the gut microenvironment provides clear evidence for APC-dependent, humoral responses elicited and matured in gut-associated lymphoid tissue and/or regional lymph nodes.^100,101^ Such microbial-antigen-containing APCs can also cross-present their phagocytosed cargo via MHC class I to induce cellular immune responses.^102,103^ Furthermore, intracellular microbes present in both cancer and immune cells^87^ can trigger direct CD8^+^ T cell responses in the tumour microenvironment (TME). Tumour neoantigen mimicry by microbial species is therefore likely to produce T cell clones of both the CD8 and CD4 lineage that are tumour-reactive despite never having previously seen actual tumour antigens. Such independent emergence of T cell populations with overlapping specificity can lead to functional immunological crosstalk that shapes overall anti-cancer immunity and influences the outcome of checkpoint-based immunotherapy.^81–84^

Currently, the net effect of such immune interaction is unclear. Generally, the gastrointestinal tract and other body surfaces are considered a rather tolerogenic environment, an idea that is consistent with the notion of productive interplay with commensals under homoeostatic conditions^104,105^ as well as the default unresponsiveness to food proteins in healthy individuals (oral tolerance).^106,107^ However, it is unknown how compartment-specific microbial tolerance influences neoantigen-directed tumour immune surveillance (even more so during perturbations such as dysbiosis or infection). Although immune tolerance to commensals and their tumour neoantigen mimics might intuitively be more associated with reduced anti-cancer immunity, evidence has shown that T cells that are specific for certain gut commensals serve immune-stimulatory functions during checkpoint-based immunotherapy.^73^ These data suggest that the ‘tolerogenic’ environment of the gut, including tolerance to neoantigen-mimicking commensals, can nevertheless support anti-cancer immunity based on yet-to-be-determined mechanisms; conceivable means include compartment-specific effects as well as temporary disruptions of epithelial barrier function leading to translocation into the systemic circulation and subsequent failure of tolerance.

In further support of clinically relevant tumour neoantigen mimicry, long-term survivors of pancreatic cancer show tumour-neoantigen-specific T cell clones with predicted cross-reactivity to microbial epitopes.^81^ In these patients, immunogenic neoantigens can be identified using a quality fitness model that takes into account differential presentation and homology to microbial-derived peptides.^81^ Of note, ‘high-quality’ neoantigens are selectively lost upon metastatic disease progression, suggesting immunoediting and, ultimately, immunological escape of clinically relevant tumour neoantigens.^81^

### Immune-modulatory activity of microbial metabolites

The microbiome-derived metabolome refers to the aggregate of individual microbial metabolites in a given compartment, several of which, including short-chain fatty acids^108,109^ and inosine,^110^ are known to have immune-modulatory activity and to influence cancer immunotherapy. Short-chain fatty acids are differentially expressed among immunotherapy responders and non-responders, and a high faecal abundance of acetic, propionic, butyric and valeric acid correlates with prolonged progression-free survival.^108,109^ The underlying mechanisms are yet to be fully elucidated, but are likely to involve altered T cell homoeostasis and differentiation as well as modulation of APC functions^108,109^ brought about by these short-chain fatty acids. Inosine as produced by *Bifidobacterium* spp. in the gut facilitates enhanced ICI treatment efficacy through a mechanism that involves the increased systemic translocation (owing to ICI-induced decreased gut barrier function) of inosine and subsequent activation of anti-cancer T cells.^110^ Considering the basic principles of immune activation and differentiation, it is very much conceivable that T cells, as well as their correspondingly induced cytokines, that recognise mimicked tumour neoantigens are subject to regulation by microbial metabolites.

## Dissecting the role of microbial tumour neoantigen mimicry

At this stage, no conclusions on the role of microbial tumour neoantigen mimicry can be drawn; mechanistic studies in mice as well as consolidation in the clinical setting are warranted. Below we provide insight into how to experimentally address microbial tumour neoantigen mimicry to better understand responsiveness to checkpoint immunotherapy and optimise patient stratification. Molecular and immunological tools are available to define the contribution of gut microbes to the responsiveness of checkpoint immunotherapy and to systematically assess whether microbial tumour neoantigen mimicry acts to foster or impede protective anti-cancer immunity.

### Generating mouse models of immunological tumour mimicry

**Fig. 2:**
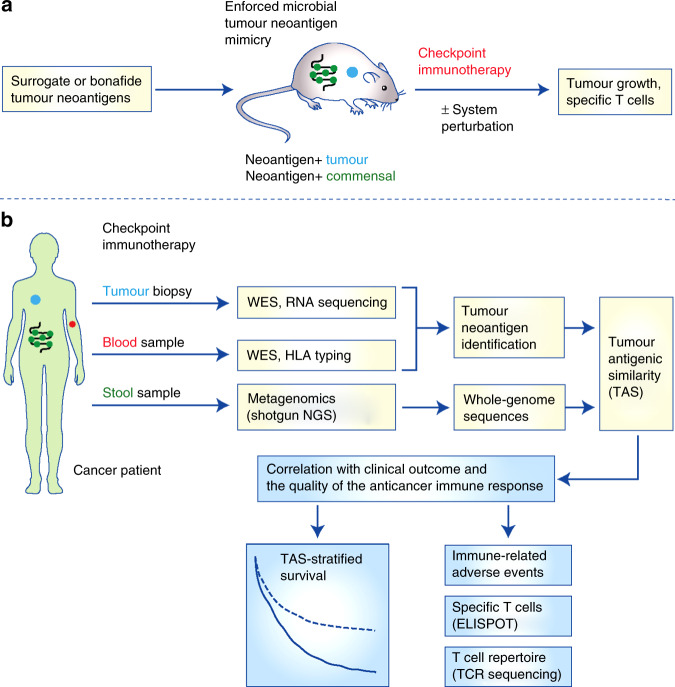
Strategies for studying microbial tumour neoantigen mimicry in mice and men. **a** Tumour cells of known neoantigen status and corresponding engineering of gut commensals can be used to generate mouse models of enforced microbial tumour neoantigen mimicry. The effects of such mimicry on checkpoint immunotherapy responsiveness and tumour growth can be investigated and the quality and strength of the anti-cancer immune response can be assessed. System perturbations such as co-treatment with antibiotics can be introduced to fine-tune the degree of tumour neoantigen mimicry in these well-defined models. **b** Using whole-exome sequencing and RNA sequencing of tumour and blood samples from cancer patients treated with immune checkpoint inhibitors, tumour neoantigens binding to respective MHC class I molecules can be identified using bioinformatics. In parallel, shotgun sequencing of stool samples can characterise the microbial metagenome and identify the antigenic sequences present in the gastrointestinal tract. Using this information, the degree of immunological similarity between tumour cells and gut microbes can be determined and summarised as ‘tumour antigenic similarity’ (TAS). Using TAS-based patient stratification, the impact of microbial tumour neoantigen mimicry on treatment response and survival can be evaluated. Companion immune profiling using methods such as enzyme-linked immunospot assay and T cell receptor sequencing can be used to corroborate clinical survival data and indicate the potential underlying mechanisms of treatment response or failure. ELISPOT enzyme-linked immunospot assay, NGS next-generation sequencing, TAS tumour antigenic similarity, TCR T cell receptor, WES whole-exome sequencing.

Modern genetic engineering facilitates the expression of almost any gene of interest in prokaryotic and eukaryotic cells and is key to mechanistically dissecting the function of genes. Using this reverse genetics approach to introduce tumour neoantigens into gut commensals, mouse models of immunological tumour mimicry can be experimentally generated. To achieve this, the genetic sequences encoding tumour neoantigens need to be cloned into suitable commensals (e.g. *Bifidobacterium*, *Bacteroides* and *Escherichia* spp.) before the genetically modified bacteria are introduced into the gastrointestinal tract of immunocompetent mice using oral gavage; sustained bacterial engraftment might involve antibiotic conditioning.^111^ Tumour neoantigen mimicry can be enforced by transplanting tumour cells into bacteria-reconstituted mice, and the impact of such mimicry on anti-cancer immunity and immunotherapy responsiveness can be investigated (Fig. 2a). Syngeneic, orthotopic tumour models are desirable, as they most closely resemble the physiological setting especially in terms of immune compatibility and an authentic TME.^112,113^ A future option might involve the use of patient-derived, orthotopic xenograft models^114^ in conjunction with human haematopoiesis-reconstituted mice (e.g. the MISTRG strain^115^), provided that limitations with adaptive immune responses can be overcome in humanised mice.^116^

The mimicked (neo-)antigens can include surrogate tumour antigens such as minigene-encoded epitopes of viral glycoproteins^117–119^ and xenogeneic proteins,^120–122^ or bona fide tumour neoantigens that arise naturally during tumour evolution. Although surrogate tumour antigen models can provide unique opportunities to track antigen-specific immune responses (which is important for initial proof-of-concept studies),^123,124^ they are largely artificial and can have limited predictive and translational value.^125,126^ Conversely, tumour neoantigens from mutagen-induced (e.g. dimethylbenzathracene, azoxymethane, *N*-ethyl-*N*-nitrosourea, UV light^127^) or genetically-engineered tumours (e.g. *Kras*^*LSL-G12D*^*; p53*^*frt/frt*^ lung adenocarcinoma,^128,129^ MMTV-PyMT^130,131^ or MMTV-neu^132^ mammary carcinoma, *Kras*^*LSL-G12D*^*; p16*^*lox/lox*^*; Pdx1-Cre* pancreatic ductal adenocarcinoma^129,133^) might be more relevant because they have evolved ‘naturally’ and gone through immune selection and immune-editing processes.^134,135^ Such engineered/autochthonous models will produce genetically heterogeneous tumours with a comparatively high frequency of protein-altering mutations and copy number variations.^136^ From such models, clonal tumour cell lines can be established, and the tumour neoantigens can be identified using next-generation sequencing at the DNA level and compared with autologous reference tissue/blood.^18^ RNA sequencing can determine the fraction of neoantigens putatively expressed, and peptide–MHC binding algorithms such as NetMHCpan^137^ can predict neoantigen immunogenicity.

### Assessing the effects of immunological tumour neoantigen mimicry on immune response and tumour growth

To investigate the functional significance of immunological tumour neoantigen mimicry for ICI responsiveness and outcome, readouts addressing the quality and strength of the anti-cancer immune response as well as changes in tumour growth are required. Flow cytometry/fluorescence-activated cell sorting can help to delineate general immune (activation) patterns in tumour tissue, blood and lymph nodes, and can further facilitate neoantigen-specific T cell tracking and purification, making use of peptide–MHC multimer technology.^119,123,138–140^ The enzyme-linked immunospot assay can probe the neoantigen-specific reactivity of ex vivo stimulated T cell populations^141,142^ and T cell receptor sequencing can characterise the clonality and overall repertoire of intratumoural T cell subsets.^143^

Complementing these important immune-targeted readouts, the effects on functional tumour growth need to be assessed, and a number of scenarios should be explored: first, ‘continuous’ tumour growth resulting from a high dose of transplanted tumour cells; second, tumour engraftment emanating from a critically low dose of transplanted tumour cells;^139,144^ and, third, ‘metastatic’ growth in a relevant compartment.^119^ These scenarios will allow various clinical settings, including those of high tumour burden, minimal residual disease and oligometastatic disease, respectively, to be experimentally explored. System perturbations such as co-treatment with antibiotics to reduce or abrogate microbial tumour neoantigen mimicry could be envisaged for control purposes; here, the particular timing of antibiotic treatment should be carefully selected considering treatment duration as well as lagging phase and washout effects.^75^

### Moving into humans…

Scrutinising and validating these preclinical results in humans are crucial for eventual clinical translation. To this end, tumour neoantigens need to be identified from tumour biopsy samples using the strategy outlined above,^18^ and the gastrointestinal microbiome composition needs to be characterised using metagenomic shotgun sequencing of stool samples.^145^ Dedicated bioinformatics interrogation can uncover the overlapping tumour neoantigen and microbial sequences, and provide an estimate for the overall ‘tumour antigenic similarity’. Correlating the degree of this similarity with clinical endpoints such as survival, treatment response and immune-related adverse events, a potential proxy for treatment efficacy,^65^ will demonstrate how microbial tumour neoantigen mimicry affects patient outcomes under ICI therapy (Fig. 2b). Complementary immune profiling using neoantigen-specific (e.g. peptide-MHC multimers, enzyme-linked immunospot assay) and -unspecific assays (e.g. flow cytometry, expression profiling, T cell receptor sequencing) will be important to identify the potentially underlying mechanisms.

## Conclusion and perspectives

Improved patient stratification and resensitisation to ICIs with appropriate drugs or interventions is key to fulfilling the potential of these immunotherapeutic agents in medical oncology. Although ICI target expression and a high TMB mechanistically facilitate and predict checkpoint immunotherapy responsiveness, they have no further therapeutic (e.g. treatment-resensitising) potential. By contrast, the composition of the gut microbiome is more dynamically regulated^146,147^ and might be an attractive target for therapeutic manipulation, given its causal role in ICI responsiveness and treatment outcome.^72–75,79^

**Fig. 3:**
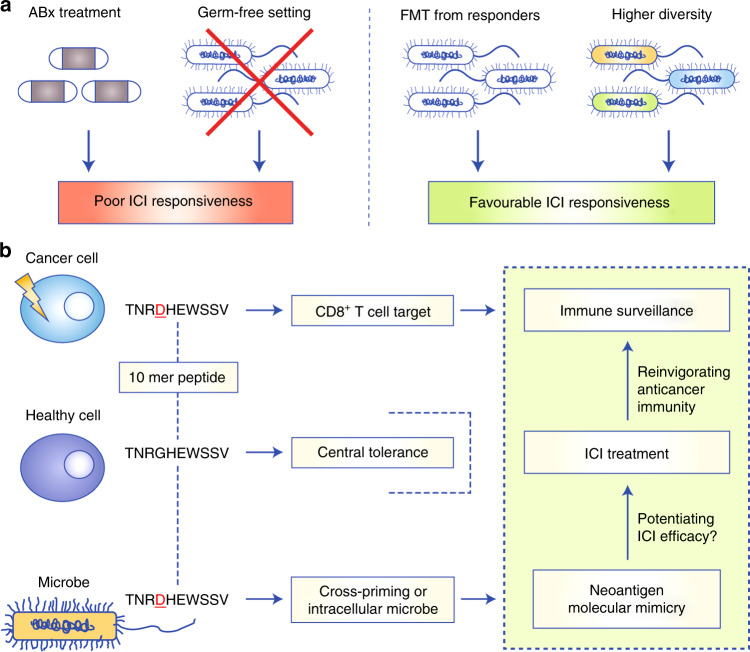
Indirect evidence suggests beneficial effects of tumour neoantigen mimicry. **a** Scenarios of reduced or absent microbial diversity (representing little/no mimicry) are associated with poor responsiveness to immune checkpoint inhibitors (ICIs), whereas scenarios of reinstated or higher microbial diversity (representing relevant/substantial mimicry) are associated with favourable ICI responsiveness. **b** Schematic showing the putative role of microbial tumour neoantigen mimicry in checkpoint-based immunotherapy. Microbial species mimicking tumour neoantigens can independently elicit tumour-reactive T cells based on antigen-presenting cell (APC) cross-priming or default APC pathways (extracellular microbes), or native ‘self’ antigen processing in infected host cells (intracellular microbes). As shown in the green box at the right-hand-side of the panel, neoantigen molecular mimicry can result in overlapping CD8^+^ (and CD4^+^) T cell specificities which might potentiate ICI therapy and contribute to protective cancer immune surveillance through reinvigoration of tumour neoantigen-specific immunity. ABx antibiotic treatment, APC antigen-presenting cell, FMT faecal microbiome transplantation, ICI immune checkpoint inhibitor.

Several mechanisms, including discrepancies in the levels of some metabolites,^148,149^ might conceivably be responsible for modulating both natural^81^ and ICI-induced^73,82–84^ anti-cancer immunity; however, evidence suggests that molecular mimicry of tumour neoantigens by microbial species is likely to be an important factor. The net effect of tumour neoantigen mimicry on ICI treatment efficacy currently remains unknown and future mechanistic and clinical studies are clearly warranted. Nonetheless, the results of various studies that have investigated microbial composition in the context of checkpoint immunotherapy responsiveness support a model in which mimicry has favourable effects on anti-cancer immunity and ICI-related treatment outcome (Fig. 3). First, co-treatment with antibiotics (which reduces potential mimicry) and organism-wide germ elimination (which precludes mimicry) are well-known to curtail the efficacy of checkpoint-based immunotherapy^73–75,79,150^—with the limitation that clinical data are derived almost exclusively retrospectively, as prospective testing of an ICI–antibiotic combination is not really feasible due to ethical concerns.^77^ Second, a higher microbial diversity index (which increases the statistical odds for mimicry) is associated with immune activation and long-term survival of pancreatic cancer patients^151^ as well as a memory T cell signature and favourable response to PD-1-targeted immunotherapy in patients with NSCLC.^152^ Along these lines, the beneficial effects of therapeutic FMT from ICI-responding patients^79^ might be at least partially attributable to an increase in the overall microbial diversity, similar to the results seen for FMT in patients with inflammatory bowel disease.^153,154^

Assuming a protective role for microbial tumour neoantigen mimicry, interventions aimed at supplementing probiotics and/or increasing microbial diversity represent rational therapeutic approaches that warrant clinical investigation.^85^ Importantly, the gut microbiome engages in crosstalk with the tumour microbiome such that therapeutic FMT will automatically also modulate the tumour microbial composition.^151^ Clinical research will show whether such interventions are sufficient to reinstate relevant tumour neoantigen mimicry or whether targeted engineering of commensals based on the tumour neoantigen landscape would be required. Such personalised, molecularly informed approaches are technically feasible, and could be achieved within a clinically reasonable time frame (approximately 6–8 weeks), provided that trial data can overcome safety concerns regarding the use of genetically-modified live bacteria.

Another promising anti-cancer strategy is the use of ICIs together with tumour-neoantigen-specific vaccines.^18,141^ Given the clarification of the definite role of tumour neoantigen mimicry in ICI responsiveness, this knowledge can help to complement MHC class I binding prediction^137^ and assist in the consideration of which neoantigens to choose for vaccination. In this way, the anti-cancer immune response could be (re-)directed specifically to molecularly mimicked neoantigens, provided that mimicry indeed shows beneficial effects on ICI treatment efficacy.

Immunogenic cancer cells adapt to the selective pressure of the immune system, conceptually traversing through three successive phases—elimination, equilibrium and escape^135,155^—by the process of immunoediting. Immunoediting of tumour neoantigens has been reported, and is associated with metastatic progression in pancreatic cancer.^81^ Other prominent mechanisms of cancer immune escape include somatic loss of HLA class I molecules (loss of heterozygosity (LOH))^156^ and defects in the MHC class I antigen processing and presenting machinery.^157^ Importantly, HLA class I LOH predicts poor overall survival in a subgroup of ICI-treated NSCLC patients and can be used as a complementary marker for checkpoint immunotherapy responsiveness to refine TMB-based stratification.^158^ In the context of tumour neoantigen mimicry and associated therapeutic interventions, HLA class I LOH needs to be considered as a potential confounder for tumour-microbiome cross-reactive T cell responses. While HLA class I LOH occurs spontaneously and is not preventable per se, any neoantigen-directed therapeutic intervention should ensure coverage of several HLA class I alleles to avoid dependence on a single genetic locus.

This review article has highlighted the crucial importance of tumour neoantigens for cancer immunotherapy and provided impetus for investigating the significance of microbial tumour neoantigen mimicry for checkpoint immunotherapy responsiveness and outcome. Immunologically informed considerations on ICI therapeutic use will optimise their clinical performance and set the stage for microbiome-targeted interventions to boost response rates and prolong survival.

## Data Availability

Not applicable.
